# Supplementary material for: Bridging Histology and Tractography: First Visualization of the Short-Range Prefrontal Connections in the Human Brain
Source: bioRxiv. 2025 Oct 22:2025.10.22.683760. Preprint. [Version 1] doi: 10.1101/2025.10.22.683760 (PMC12633442; doi:10.1101/2025.10.22.683760)
Supplement: 1 [file NIHPP2025.10.22.683760V1-supplement-1.pdf]

bioRxiv preprint doi: https://doi.org/10.1101/2025.10.22.683760; this version posted October 23, 2025. The copyright holder for this preprint (which was not certified by peer review) is the author/funder, who has granted bioRxiv a license to display the preprint in perpetuity. It is made available under aCC-BY 4.0 International license.

6. Supplementary Figures

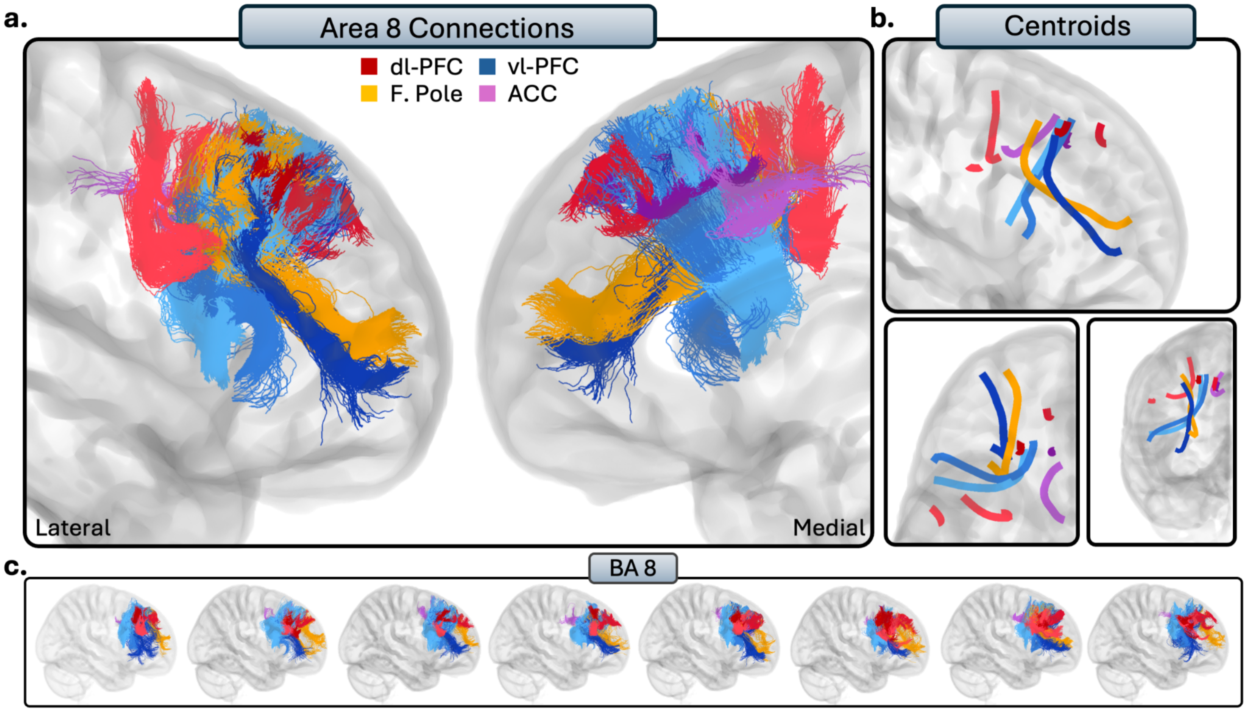

Supplementary Figure 1 - As the connections of area 8 are volumetrically large, we separated its visualization from the dl-PFC (Figure 3). a) The connections of area 8 to the rest of the PFC. Red = tracts to the dl-PFC, blue = vl-PFC, orange = frontal pole, and purple = ACC. b.) Overall trajectories of the short fibers. Top: Sagittal view, left: axial view, right: coronal view. c.) Individual subject variability for each prefrontal region.

bioRxiv preprint doi: https://doi.org/10.1101/2025.10.22.683760; this version posted October 23, 2025. The copyright holder for this preprint (which was not certified by peer review) is the author/funder, who has granted bioRxiv a license to display the preprint in perpetuity. It is made available under aCC-BY 4.0 International license.

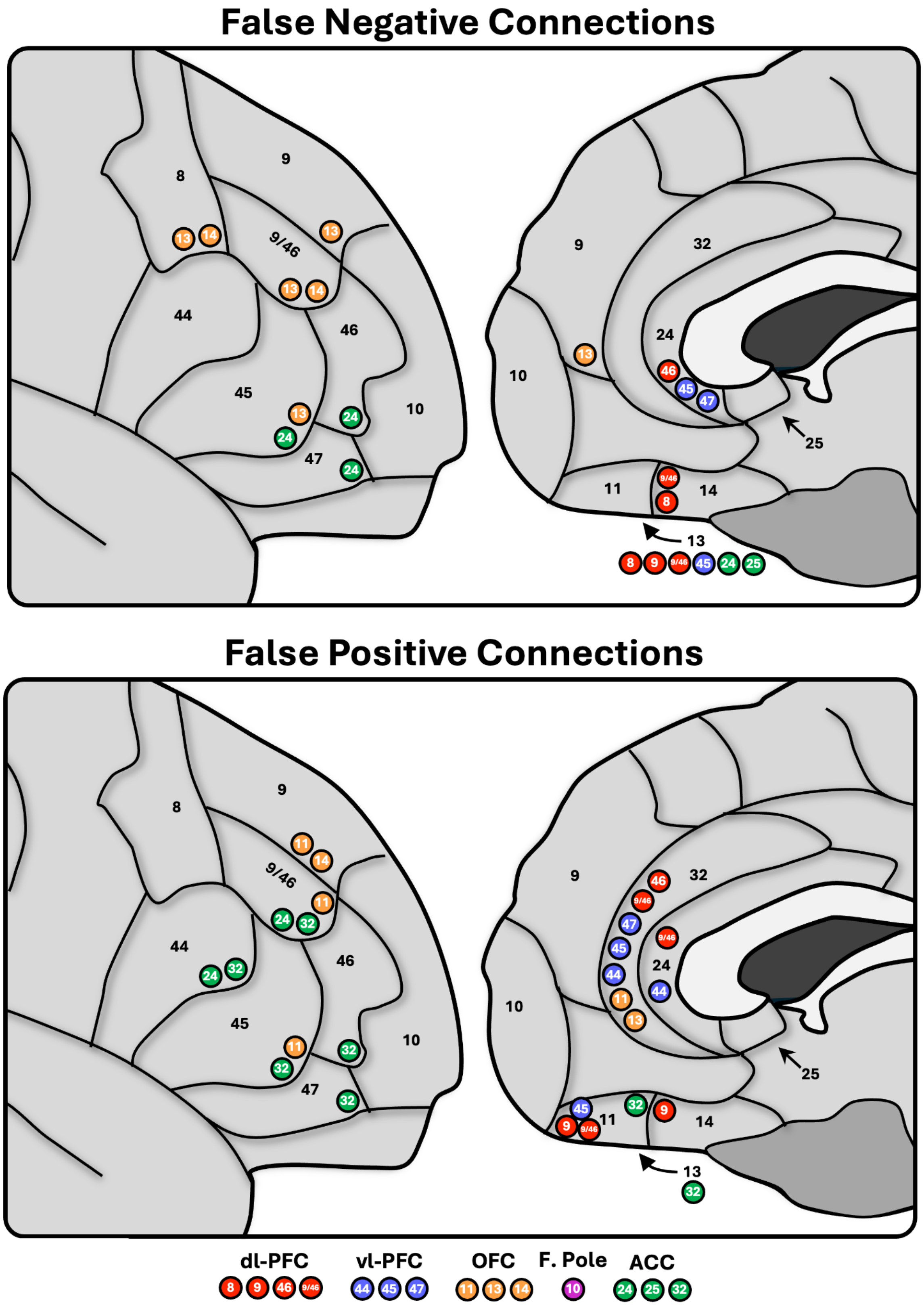

bioRxiv preprint doi: https://doi.org/10.1101/2025.10.22.683760; this version posted October 23, 2025. The copyright holder for this preprint (which was not certified by peer review) is the author/funder, who has granted bioRxiv a license to display the preprint in perpetuity. It is made available under aCC-BY 4.0 International license.

Supplementary Figure 2 - Schematic overview of the False Negative (FN) and False Positive (FP) tractography results. These are the prefrontal regions where our tractography differed the most from histological findings. Notably, most of these were present in the OFC and ACC.
